# FTIR Characterization of Sulfated Polysaccharides Obtained from *Macrocystis integrifolia* Algae and Verification of Their Antiangiogenic and Immunomodulatory Potency In Vitro and In Vivo

**DOI:** 10.3390/md21010036

**Published:** 2022-12-31

**Authors:** Marilú Roxana Soto-Vásquez, Paúl Alan Arkin Alvarado-García, Fadia S. Youssef, Mohamed L. Ashour, Hanin A. Bogari, Sameh S. Elhady

**Affiliations:** 1 Facultad de Farmacia y Bioquímica, Universidad Nacional de Trujillo, Av. Juan Pablo II, Trujillo 13011, Peru; 2 Escuela de Medicina, Universidad Cesar Vallejo, Av. Larco s/n, Trujillo 13011, Peru; 3 Department of Pharmacognosy, Faculty of Pharmacy, Ain-Shams University, Abbasia, Cairo 11566, Egypt; 4 Pharmacy Program, Department of Pharmaceutical Sciences, Batterjee Medical College, Jeddah 21442, Saudi Arabia; 5 Department of Pharmacy Practice, Faculty of Pharmacy, King Abdulaziz University, Jeddah 21589, Saudi Arabia; 6 Department of Natural Products, Faculty of Pharmacy, King Abdulaziz University, Jeddah 21589, Saudi Arabia

**Keywords:** algae, *Macrocystis integrifolia*, anti-allergic, antiangiogenic, polysaccharides, public health, drug discovery

## Abstract

The aim of this study was to evaluate the antiangiogenic and immunomodulatory potential of sulfated polysaccharides from the marine algae *Macrocystis integrifolia* characterized by FTIR. The cytotoxicity of sulfated polysaccharides was evaluated using the 3-(4,5-dimethylthiazol-2-yl) -2,5-diphenyltetrazolium bromide (MTT) assay. Antiangiogenic activity was evaluated using the chicken chorioallantoic membrane (CAM) assay. Immunomodulatory activity was determined on macrophage functionality and allergic response. The results showed that sulfated polysaccharides significantly decreased angiogenesis in chicken chorioallantoic membranes (*p* < 0.05). Likewise, they inhibited in vivo chemotaxis and in vitro phagocytosis, the transcription process of genes that code the enzymes cyclooxygenase-1 (COX-1), cyclooxygenase-2 (COX-2) and nitric oxide synthase-2 (NOS-2) and the nuclear factor kappa-light chain enhancer of activated B cells (NF-κB), showing immunomodulatory properties on the allergic response, as well as an in vivo inhibitory effect in the ovalbumin-induced inflammatory allergy model (OVA) and inhibited lymphocyte proliferation specific to the OVA antigen in immunized mice. Finally, these compounds inhibited the histamine-induced skin reaction in rats, the production of immunoglobulin E (IgE) in mice, and the passive response to skin anaphylaxis in rats. Therefore, the results of this research showed the potential of these compounds to be a promising source for the development of antiangiogenic and immunomodulatory drugs.

## 1. Introduction

Cancer affects humanity and is currently considered to be one of the main global public health problems. In 2020, it resulted in 1.4 million deaths, 47% of them aged 69 years or younger. The number of cancer cases in the Region of the Americas was estimated to be 4 million in 2020 and is expected to increase up to 6 million by 2040 [1,2]. However, the lack of specificity of anticancer drugs, their harmful side effects, and the appearance of resistance to chemotherapy, led to the appearance of new treatment strategies, such as antiangiogenic therapy. This was based on the inhibition of tumor vascularization (tumor angiogenesis) in order to prevent the formation of new blood vessels that promote tumor development and metastasis [3].

Additionally, malfunctions in the immune system are among the main etiologies responsible for the development of autoimmune diseases, rejection of transplanted organs, infectious diseases, and even cancer, generating high rates of morbidity and mortality and high social costs [4]. Such reasons have aroused great interest in the scientific community for the study of immunomodulation, with the aim of preventing diseases, rather than treating them in their already advanced state. In addition, numerous analyses in cancer survivor patients have identified the activation of certain immunological patterns related to the phenomenon of immunosurveillance, which would activate certain adaptive mechanisms to develop antibodies that inhibit the “stop” signals that cancers generate to prevent T cells from acting against them [5].

Therefore, antiangiogenic and immunomodulatory activity are the two main strategies that are adopted for the development of new anticancer drugs at present. Recently, marine organisms have been considered as an everlasting source of medicinally valuable drugs [6,7,8,9]. In recent years, the scientific community has focused its attention on the study of sulfated polysaccharides from marine algae, due to their numerous and promising pharmacological properties [10]. Algae, as a marine natural product, represent one of the sources for the development of new anticancer drugs. Algae constitute 90% of oceanic biomass and are rich in polyphenols and sulfated polysaccharides (PS); many of them showed cytotoxic and antitumor activities [11]. In this sense, previous research showed that sulfated polysaccharides from algae of the genus *Sargassum* revealed antiangiogenic and antiproliferative effects [10]. Similarly, fucoidan, a sulfated polysaccharide, previously showed potent in vitro and in vivo inhibition of angiogenesis in addition to a reduction in metastasis in different types of cancer, as well as causing apoptosis and activation of the immune system [12]. Furthermore, sulfated polysaccharides also exerted antiviral, antithrombotic, anticoagulant, and anti-inflammatory properties, in addition to being effective in kidney and liver disorders [13,14].

*Macrocystis integrifolia* is a form of large brown algae that can reach more than 50 m in length, with a main distribution in the southern hemisphere and on the eastern coasts of the North Pacific. The mucilage of this algae is of great importance, since it constitutes the source of alginates used in the food, cosmetic and pharmaceutical industries [15,16]. Thus, herein, we aim to isolate sulfated polysaccharides from seaweed *Macrocystis integrifolia,* followed by their profiling using Fourier transform infrared (FTIR) spectroscopic analysis. The antiangiogenic was evaluated using chorioallantoic membrane (CAM) assay, whereas the immunomodulatory potential of sulfated polysaccharides was performed both in vitro and in vivo, followed by the assessment of different parameters to consolidate the results, such as mRNA levels of NOS-2, COX-1, COX-2, NF-κB and IκB. Furthermore, antiallergic potential was determined using ovalbumin (OVA)-induced allergic inflammation in vivo model and histamine skin reaction, in addition to the determination of a specific IgE in OVA-immunized mice.

## 2. Results and Discussion

### 2.1. Fourier Transform Infrared (FTIR) Spectroscopic Analysis

**Figure 1:**
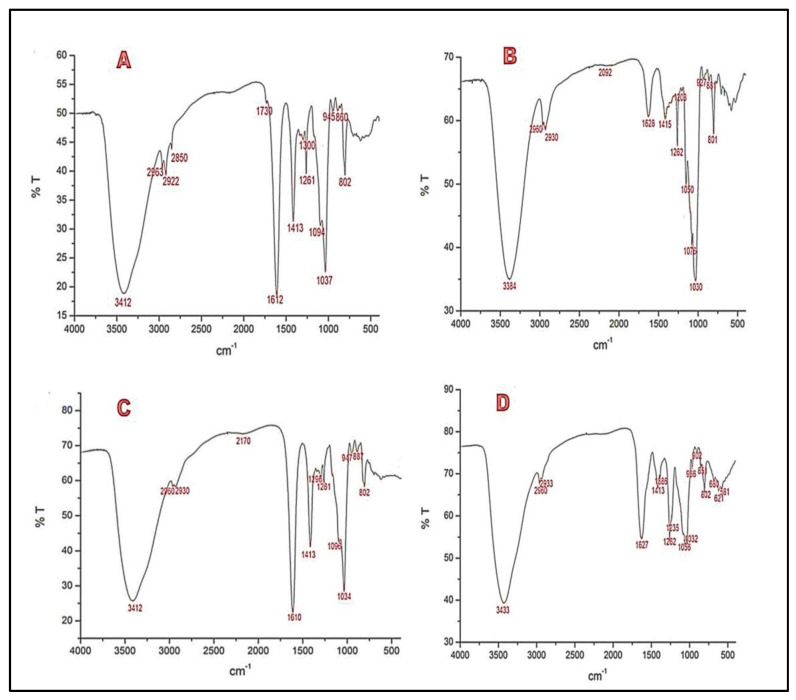
FTIR spectra of sulfated polysaccharide fractions from *M. integrifolia*. (**A**) Fraction MIF1, (**B**) Fraction MIF2, (**C**) Fraction MIF3, (**D**) Fraction MIF4.

The FTIR spectra of the fractions of *M. integrifolia* were shown in Figure 1, where it is evident that the absorption bands of the spectra of the fractions MIF1 (3412 cm^−1^), MIF2 (3384 cm^−1^), MIF3 (3415 cm^−1^), and MIF4 (3433 cm^−1^) correspond to the O-H bonds; and the absorption bands of the spectra of the fractions MIF1 (2963 cm^−1^, 2922 cm^−1^, 2850 cm^−1^), MI F2(2960 cm^−1^, 2930 cm^−1^), MIF3 (2960 cm^−1^, 2930 cm^−1^), and MIF4 (2960 cm^−1^, 2833 cm^−1^) are related to the C-H stretching of the pyranoid rings. The intense bands around 1700 cm^−1^, 1600 cm^−1^ in the spectra of fractions MIF1(1612 cm^−1^), MIF2 (1628 cm^−1^), MIF3 (110 cm^−1^) and MIF4 (1627 cm^−1^) are attributed to the stretching vibration of the carboxyl or acetyl group bonds (C=O), due to the presence of uronic acids, which mainly derived from the residues of guluronic and mannuronic acid (C=O) [17]. Likewise, the MIF2 and MIF4 fractions are of very low intensity, which suggests that there is only a low quantity. The absorption of the bands around 1270–1220 cm^−1^ of the spectra of the fractions MIF1 (1261 cm^−1^), MIF2 (1262 cm^−1^), MIF3 (1261 cm^−1^) and MIF4 (1262 cm^−1^), is related to the asymmetric stretching of the vibration of the sulfate group (S=O), a characteristic component of fucoidans [18,19]. The MIF3 fraction presents a very low intensity, which postulated its existence in a low quantity. Likewise, the MIF4 fraction (581 cm^−1^), presents an additional band of symmetrical stretching vibration of the sulfate group at around 600–570 cm^−1^.The bands around the 1076–1030 cm^−1^ of the fractions MIF1 (1037 cm^−1^), MI F3 (1076 cm^−1^, 1050 cm^−1^, 1030 cm^−1^), MI F4 (1056 cm^−1^, 1032 cm^−1^), correspond to symmetrical stretching vibrations characteristic of the hemiacetal bond of the sugar ring (C-O-C). In the MIF3 fraction, more absorption peaks are observed compared to the other fractions, indicating that it presents more types of sugar.

### 2.2. Chemical Composition of M. integrifolia Polysaccharide Fractions

The main components of brown algae are polysaccharides, where the sulfated polysaccharides constitute a very common type of structural polysaccharide, based mainly on sulfated L-fucose (Fuc), with less than 10% of the other monosaccharides such as mannose (Man), galactose (Gal), xylose (Xyl), glucose (Glu), rhamnose (Rha) and uronic acids (UA) [20,21,22]. Table 1 shows the chemical composition of the different fractions of sulfated polysaccharides from *M. integrifolia*. The M1F1 fraction contains six sugars: 88.5% Fuc, 3.6% Xyl, 4.1% Man, 1.2% Gal, 1.5% Glu and 1.1% Rha; 3.8% UA and 33.2% sulfates, with a molecular weight (Wm) of 49.2 (kDa). The M1F2 fraction presents the same sugars as M1F1 but differs in percentages (88.1% Fuc, 3.8% Xyl, 4.9% Man, 1.8% Gal, 0.6% Glu, 0.8% Rha). The content of 5.1% UA and 27.2% sulfates is below that of M1F1, and its molecular weight is 62.1 kDa. When comparing these two fractions with other investigations, they are observed to have the same compounds of GA-fucoidan type Fucoidan [23]. Similarly, the MIF3 fraction is composed of two sugars, such as fucose (87.1%) and mannose (12.9%), in addition to UA (4.8%) and sulfates (26.8%), with a molecular weight of 70.8 kDa; therefore, it could be fucoidan type G-fucoidan [23]. Regarding the MIF4 fraction, it only presents fucose (100%) in addition to UA (4.1%) and sulfates (25.1%), with a molecular weight of 88.4 kDa, which is why it resembles F-fucoidan [23]. Similarly, the M1F1 fraction has the lowest molecular weight and the highest sulfate content compared to the other fractions (*p* < 0.05). Gupta et al. [24] reported that the molecular weights of fucoidans fall into three classes: low-molecular-weight fucoidans (LMWF) (10–50 kDa), medium-molecular-weight fucoidans (MMWF) (50–100 kDa), and high-molecular-weight fucoidans (HMWF)(>100 kDa). When comparing these molecular weights with the MIF1 fraction (49.2 kDa), M1F2 (62.1 kDa), MIF3 (70.8 kDa) and MIF4 (88.4 kDa), MIF1 would be classified within the low-molecular weight-fucoidans, while the M1F2 fractions, M1F3 and M1F4, would be classified as having medium molecular weight.

### 2.3. Evaluation of Antiangiogenic Activity Using Chorioallantoic Membrane (CAM) Assay 

In vivo CAM assay is a very effective and popular method to evaluate the antiangiogenic potency of natural products such as plant extracts. In addition, it is an alternative animal model that is adopted to screen different substances comprising cosmetics or natural products such as essential oils, sulphated polysaccharides, or extracts [25]. The probable antiangiogenic effect of the four sulfated polysaccharide fractions MIF1, MIF2, MIF3 and MIF4 was determined using different concentrations for each, namely, 10, 50 and 100 μg/mL, in addition to the normal control. CAM is a vascular chick membrane that is composed of two mesodermal layers: chorionic and allantois epithelium. CAM transverse sections displayed allantoic epithelium, chorionic epithelium showing small blood vessels and mesenchyme revealing medium-size blood vessels [26].

**Figure 2:**
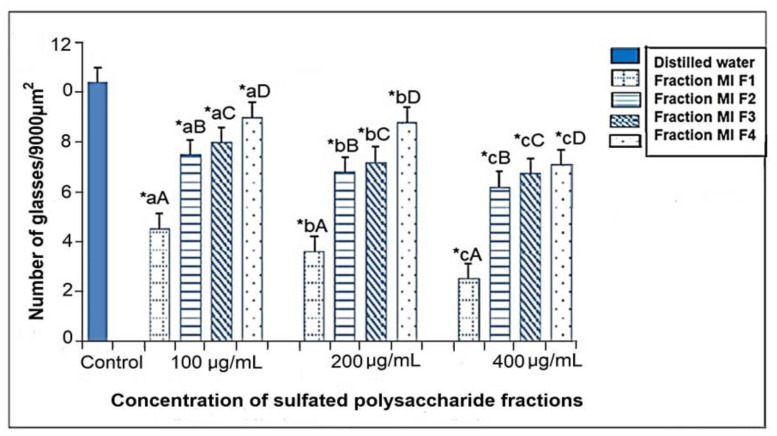
Microvascular densities at 9000 μm^2^ of CAM of the different fractions of sulphated polysaccharides of seaweed *Macrocystis integrifolia;* Data are presented as mean ± SD (*n* = 3). * indicates statistically significant differences (*p* < 0.05) between the treatments and the control, determined with the ANOVA statistic. Lowercase letters indicate statistical differences between the concentrations of the same fraction and uppercase letters indicate the statistical differences between fractions (*p* < 0.05) with the Tukey multirank test.

The microvascular density analysis illustrated that the number of vessels/9000 μm^2^ on CAM was 10.4 ± 0.15 for control, 4.8 ± 0.2, 7.2 ± 0.25, 8.0 ± 0.15 and 9.2 ± 0.2 for MIF1, MIF2, MIF3 and MI F4 at 100 μg/mL, respectively. At 200 μg/mL, values ranged between 3.6 ± 0.3, 6.8 ± 0.25, 7.2 ± 0.1 and 8.8 ± 0.15, respectively. Furthermore, in MIF1, MIF2, MIF3 and MI F4 revealed 2.5 ± 0.32, 6.2 ± 0.2, 6.9 ± 0.33 and 7.5 ± 0.4 to be the number of vessels/9000 μm^2^ on CAM at 400 μg/mL. Thus, from the results illustrated in Figure 2, it was clear that the studied fractions showed antiangiogenic activity in vivo in the chicken chorioallantoic membrane (CAM) model, showing a statistically significant decrease (*p* < 0.05) in the number of average blood vessels, where the MIF1 fraction showed the most promising activity at all tested doses. This coincides with previous publications verifying the use of the same model that sulfated polysaccharides decrease capillary density in vivo in addition to inhibiting tubulogenesis in rabbit aortic endothelial cells (RAEC), further consolidating the antiangiogenic effect of these compounds [27,28]. Likewise, the MIF1 fraction presents a higher percentage of sulfates (33.2%) and a lower molecular weight (49.2 kDa) compared to the other fractions. Previous studies noted that brown algae fucoidans possess various biological activities, including antiangiogenic activities [29,30], and this could be attributed to the degree of sulfation, which significantly influences the antiangiogenic activity level of fucoidans [31], which, in turn, could significantly suppress the mitogenic and chemotactic actions of vascular endothelial growth factor 165 (VEGF_165_) by preventing VEGF_165_ from binding to its cell surface receptor [32]. Furthermore, various investigations mentioned that fucoidans with MW > 30 kDa and a high degree of sulfation have an antiangiogenic effect [33,34].

### 2.4. Determination of Cytotoxicity by MTT Assay

**Figure 3:**
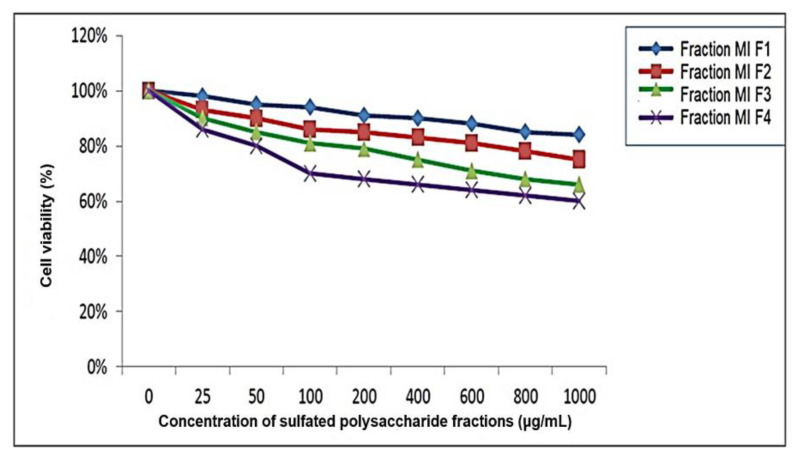
Percentage of cell viability of fractions of sulfated polysaccharides from seaweed *Macrocystis integrifolia.*

In the present study, the cell viability of fractions of sulfated polysaccharides from *Macrocystis integrifolia* algae (fractions MIF1, MIF2, MIF3 and MIF4) was evaluated using the MTT method in secondary cultures of chicken embryo fibroblasts (SCEFP) (Figure 3). The MIF1 fraction was observed to have a higher percentage of cell viability than all fractions at tested concentrations ranging from 25 to 1000 μg/mL, which coincides with other studies, where it is observed that sulfated polysaccharide compounds do not present significant toxicity in vitro, further reflecting their relative safety [12].

### 2.5. Evaluation of the Immunomodulatory Effect of Sulfated Polysaccharides from Seaweed M. integrifolia on the Functionality of Peritoneal Macrophages

#### 2.5.1. In vivo Cellular Chemotaxis Assay

**Figure 4:**
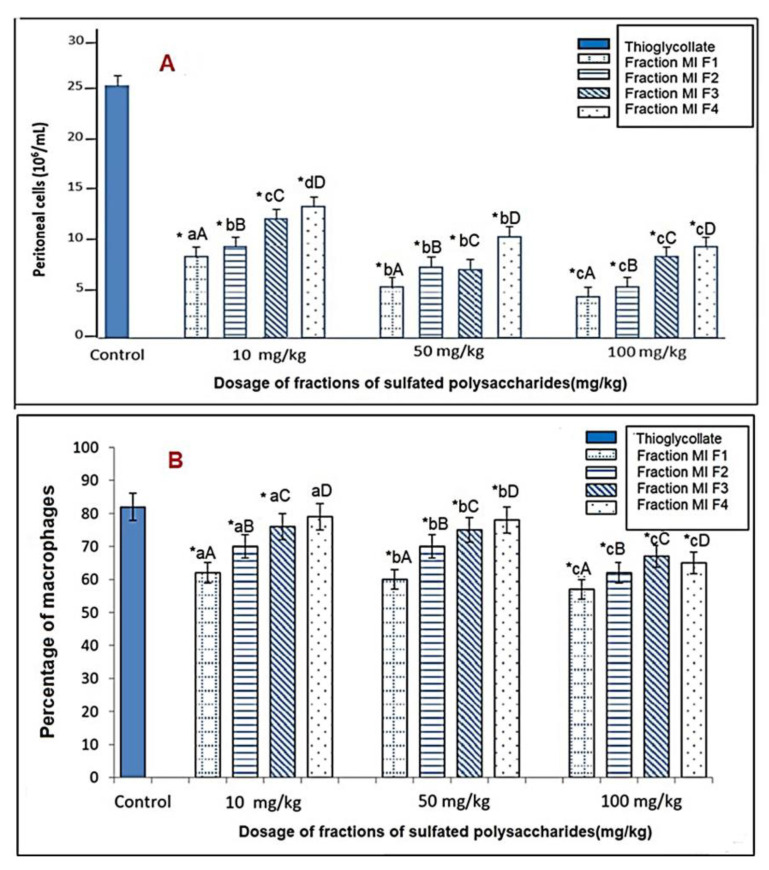
Effect of sulfated polysaccharide fractions on the cell count of the peritoneal exudate of rats; five days after intraperitoneal administration of thioglycolate. (**A**) Total number of cells in the peritoneal exudate. (**B**) Percentage of macrophages in the total cell population. Values represent the mean ± SD (*n* = 3). * indicates statistically significant differences (*p* < 0.05) between the treatments and the control, determined with the ANOVA statistic. Lowercase letters indicate statistical differences between the concentrations of the same fraction and uppercase letters indicate statistical differences between fractions (*p* < 0.05) with the Tukey multi-rank test.

Thioglycolate-induced peritonitis (TIP) is a well-established model that was adopted to evaluate the acute chemotactic responses of immune cells to the peritoneal cavity [35,36], reaching an accumulation of 25 × 10^6^ cell/mL (Figure 4A), where more than 80% of the cell population was macrophages (Figure 4B). As a result of the *ip* administration of the sulfated polysaccharide fractions at 10, 50 and 100 mg/kg, a significant reduction (*p* < 0.05) in the total number of cells that migrated to the peritoneum in response to the inflammatory stimulus was evidenced (Figure 4A). It was clear that MIF1, MIF2, MIF3, and MIF4 reduced the total number of cells in the peritoneal exudate by 66%, 59%, 48% and 44%, respectively, at a dose of 10 mg/kg, compared to the control group that received thioglycolate. Meanwhile, at a dose of 50 mg/kg, they showed a reduction of 76%, 68%, 72%, and 46%, respectively, in the total number of cells in the peritoneal exudate relative to the control group. However, the greatest reduction was observed at a dose of 100 mg/kg, where MIF1, MIF2, MIF3, and MIF4 showed a reduction of 83%, 79%, 68%, 60%, respectively, relative to the control group (Figure 4A). The reduction in the number of total peritoneal cells corresponded to a reduction in the number of macrophages, since the proportion of these in the total cell population remained constant (Figure 1B); that is, the reduction was not at the expense of a population of peritoneal cells other than macrophages. The illustrated results showed that the MIF1 fraction showed the greatest inhibition of cellular chemotaxis with statistically significant differences (*p* < 0.05), evidenced by the reduced number of cells in the peritoneal exudate, as well as the reduced percentage of macrophages in the total cell population. It should be noted that macrophages play an important role in the immune response, so overstimulation could cause tissue failure and even necrosis [37]. Sulfated polysaccharides such as fucoidans were investigated in experimental models, showing immunomodulatory effects [38,39]. Indeed, fucoidan from *F. vesiculosus* induces macrophage activation [40]. Furthermore, fucoidan of *Undaria pinnatifitida*, an edible seaweed, may increase inflammation by increasing the production of pro-inflammatory cytokines [41]. In this sense, the evidence affirms a relationship between the structure and immunomodulatory effects of fucoidan, where low-molecular-weight fucoidan presents more favorable bioactivity in vitro and in vivo studies compared to high-molecular-weight fucoidan [42,43,44].

#### 2.5.2. In Vitro Assay to Determine Phagocytic Activity

**Figure 5:**
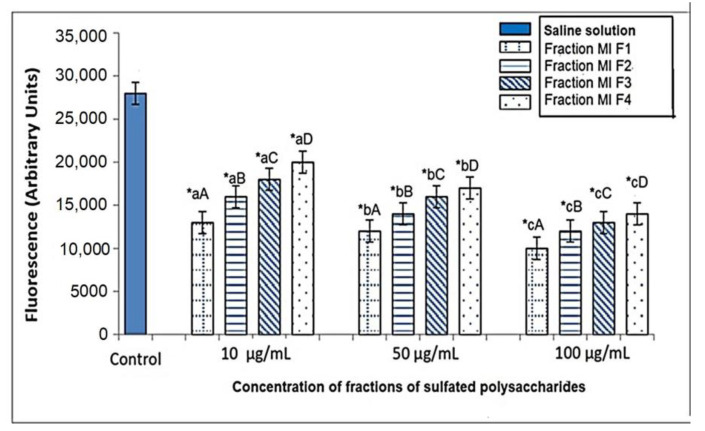
In vitro effects of *M. integrifolia* sulfated polysaccharide fractions on the phagocytosis of *Kluyveromyces lactis* cells by rat peritoneal macrophages. Values represent the mean ± SD (*n* = 3). The * indicates statistically significant differences (*p* < 0.05) between the treatments and the control, determined with the ANOVA statistic. Lowercase letters indicate statistical differences between the concentrations of the same fraction and uppercase letters indicate statistical differences between fractions (*p* < 0.05) with the Tukey multi-rank test.

Phagocytosis is one of the main functions of macrophages and, to a certain extent, reflects the state of immune function within the body. They can remove cellular debris and pathogenic organisms to maintain body homeostasis. Previous studies have shown that sulphated polysaccharides can effectively control immunity by regulating macrophage phagocytosis [45]. The results illustrated in Figure 5 showed that different fractions of sulfated polysaccharides MIF1, MIF2, MIF3 and MIF4 inhibited phagocytic activity in a dose-dependent manner relative to the control group, which received normal saline. MIF1, MIF2, MIF3 and MIF4 reduced cell phagocytosis by 47.9%, 41.1%, 33.9% and 21.4% respectively at a dose of 10 mg/kg compared to the control group, while, at a dose of 50 mg/kg, they showed reductions of 53.6%,49.6%,42.9% and 39.3%, respectively, in cell phagocytosis with respect to the control group. However, the greatest reduction in cell phagocytosis was observed at a dose of 100 mg/kg, where MIF1, MIF2, MIF3 and MIF4 showed 64.3%, 57.1%, 50.8% and 48.2% reductions, respectively, compared to the control group (Figure 5). The results presented in Figure 5 also highlighted that the MIF1 fraction showed the most significant potency in the inhibition of cell phagocytosis, which coincides with the work of Tavarsa et al., who showed that sulphated polysaccharides could improve the inflammatory condition of macrophages through the inhibition of phagocytosis [28]. In addition, a study indicated that excess phagocytosis decreased after treatment with *Fucus vesiculosus* fucoidan, and that this compound could regulate phagocytosis by modulating TNF-α production [46].

### 2.6. Determination of the Expression of Genes Encoding Enzymes, Cytokines and Immune Response Transcription Factors

#### Determination of mRNA Levels of NOS-2, COX-1, COX-2, NF-κB and IκB

**Figure 6:**
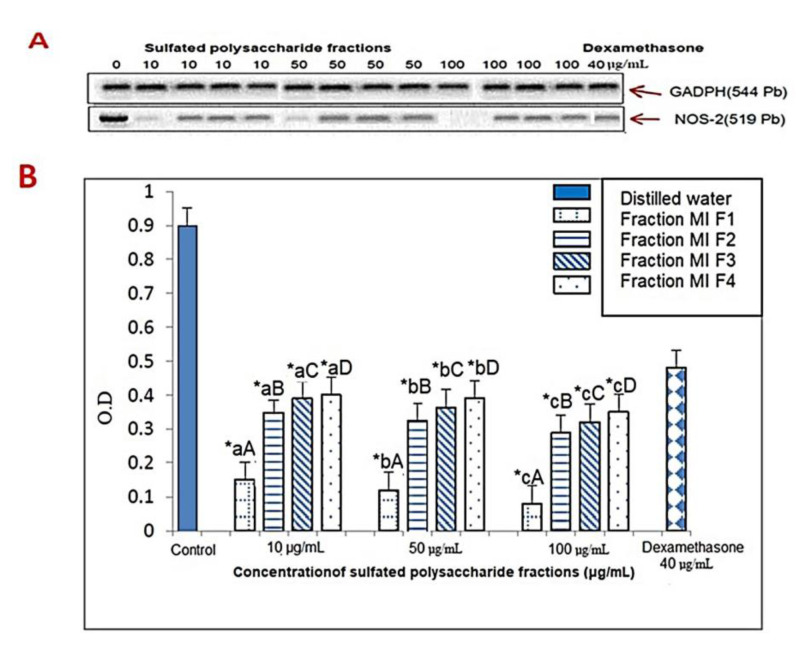
In vitro effects of sulfated polysaccharide fractions obtained from *M. integrifolia* algae and dexamethasone on NOS-2 mRNA levels in murine peritoneal macrophages stimulated with LPS and IFNγ, determined by RT-PCR. (**A**) Analysis of the RT-PCR products in the 2% agarose gel; (**B**) mRNA levels quantified by densitometric analysis, in all cases, expressed with respect to the value obtained for GADPH on the same gel. The bars show the mean ± SD of [DO band NOS-2/DO band GADPH], DO optical density] (*n* = 3). The * indicates statistically significant differences (*p* < 0.05) between the treatments and the control, determined with the ANOVA statistic. Lowercase letters indicate statistical differences between the concentrations of the same fraction and uppercase letters indicate statistical differences between fractions (*p* < 0.05) with the Tukey multi-rank test.

NO production requires nitric oxide synthase (NOS), where various inflammatory stimuli can trigger the expression of inducible NOS (iNOS), in different types of cells, such as macrophages [47]. Here, NO production was evaluated by RT-PCR determination of iNOS mRNA expression after the addition of sulfated polysaccharide fractions MIF1, MIF2, MIF3 and MIF4 at different doses, namely, 10, 50 and 100 μg/mL. The results illustrated in Figure 6 showed that the sulfated polysaccharides of different fractions significantly reduced (*p <* 0.05) the mRNA expression levels in a dose-dependent manner, which, when produced in excess, can cause diseases such as rheumatoid arthritis, diabetes mellitus, atherosclerosis, as well as septic shock [48,49]. At a dose of 10 μg/mL, MIF1, MIF2, MIF3 and MIF4 reduced iNOS mRNA expression, as evidenced by the reduction in optical density by 83%, 59%, 58%, and 56%, respectively, relative to the control group. Meanwhile, at 50 μg/mL, they showed 88%, 61%, 60% and 57% reductions in iNOS mRNA expression, respectively, compared to the control group, while the highest reduction was observed at 100 μg/mL, showing 92%, 68%, 65% and 63% with respect to the control group, where MIF1 showed the highest inhibitory activity at all tested doses. This is supported by an investigation using fucoidan extracted from *Sargassum fusiforme,* where the results demonstrated the inhibition of NO production and PGE_2_ by down-regulating iNOS and COX-2 [50]. Furthermore, a fucose-rich polysaccharide fraction of *Chnoospora minima* also decreased the production of PGE_2_, TNF-α, IL−1β, IL-6, NO, iNOS, and COX-2 expression in a dose-dependent manner. This inhibition of pro-inflammatory cytokine production is a crucial mechanism to control inflammation [51].

**Figure 7:**
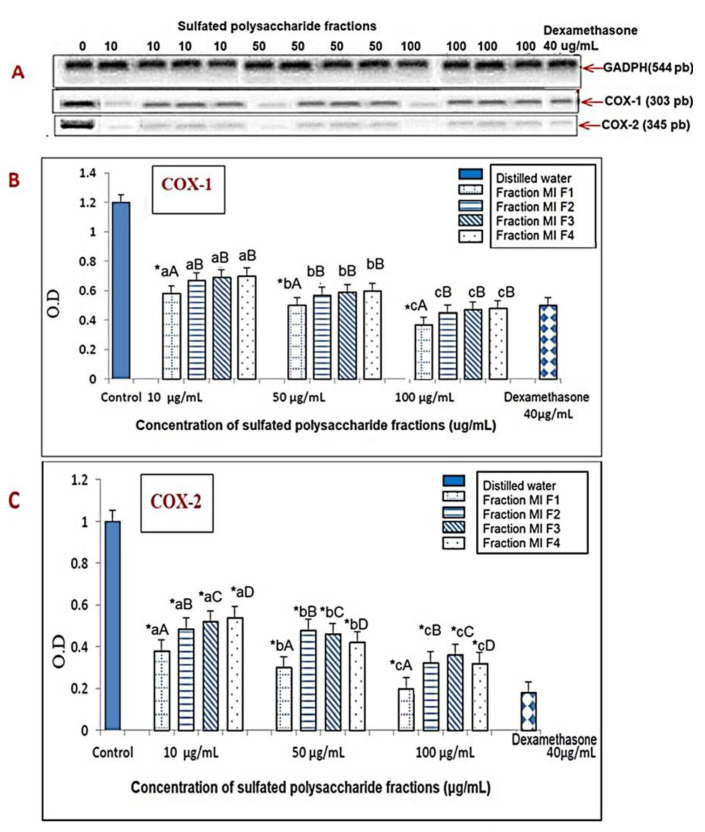
In vitro effects of *M. integrifolia* sulfated polysaccharides and dexamethasone on COX-1 and COX-2 mRNA levels in murine peritoneal macrophages stimulated by LPS and IFN-k, determined by RT-PCR. (**A**) Analysis of the RT-PCR products on 2% agarose gel; (**B**,**C**) COX-1 and COX-2 mRNA levels, respectively, quantified by densitometric analysis, in all cases expressed with respect to the value obtained for GADPH on the same gel. The bars show the mean ±SD (*n* = 3). * indicates statistically significant differences (*p* < 0.05) between the treatments and the control, determined with the ANOVA statistic. Lowercase letters indicate statistical differences between the concentrations of the same fraction and uppercase letters indicate statistical differences between fractions (*p* < 0.05) with the Tukey multi-rank test.

Similarly, the results represented in Figure 7A−C showed that the sulfated polysaccharides revealed a more selective inhibition of COX-1 and COX-2, since they inhibit the levels of COX-1 and COX-2 mRNA at concentrations of 10, 50 and 100 μg/mL, in a dose-dependent manner. Therefore, at a dose of 100 μg/mL, MIF1, MIF2, MIF3 and MIF4 reduced the expression of COX-1 mRNA, evidenced by reductions in optical density of 66.7%, 65.0%, 63.3% and 60.0%, respectively, relative to the control group, approaching that of dexamethasone, which revealed a reduction of 58.4% at 40 μg/mL. Similarly, they reduced COX-2 mRNA expression by 82.1, 65.2, 64.1, and 70.2%, respectively, relative to the control group, in a similar manner to that of dexamethasone, which revealed a reduction of 85.2% at 40 μg/mL, where MIF1 revealed the highest inhibitory potential at all examined doses. This coincides with in silico studies of fucoidan-type sulfated polysaccharides, which turned out to be potent inhibitors of COX-1 and COX-2, and their effect may be greater than that of alginates [52]. Another study showed that *Fucus vesiculosus* fucoidan showed the better inhibition of both isoforms of the COX enzyme compared to indomethacin; the inhibition of COX-2 was higher than COX1 [53]. Furthermore, fucoidan fractions with a low molecular weight and high sulfate content showed the downregulation of iNOS and COX-2 [54]. In addition, a commercial fucoidan from *Undaria pinnatifida* (with high sulfate and fucose content and unknown Mw) decreased the expression of COX-2 [55]. The evidence points to the fact that molecular weight and sulfate content play a significant role in COX inhibition [56], and fucoidan can down-regulate IL−1β-induced COX-2 expression, confirming its anti-inflammatory potential [53].

**Figure 8:**
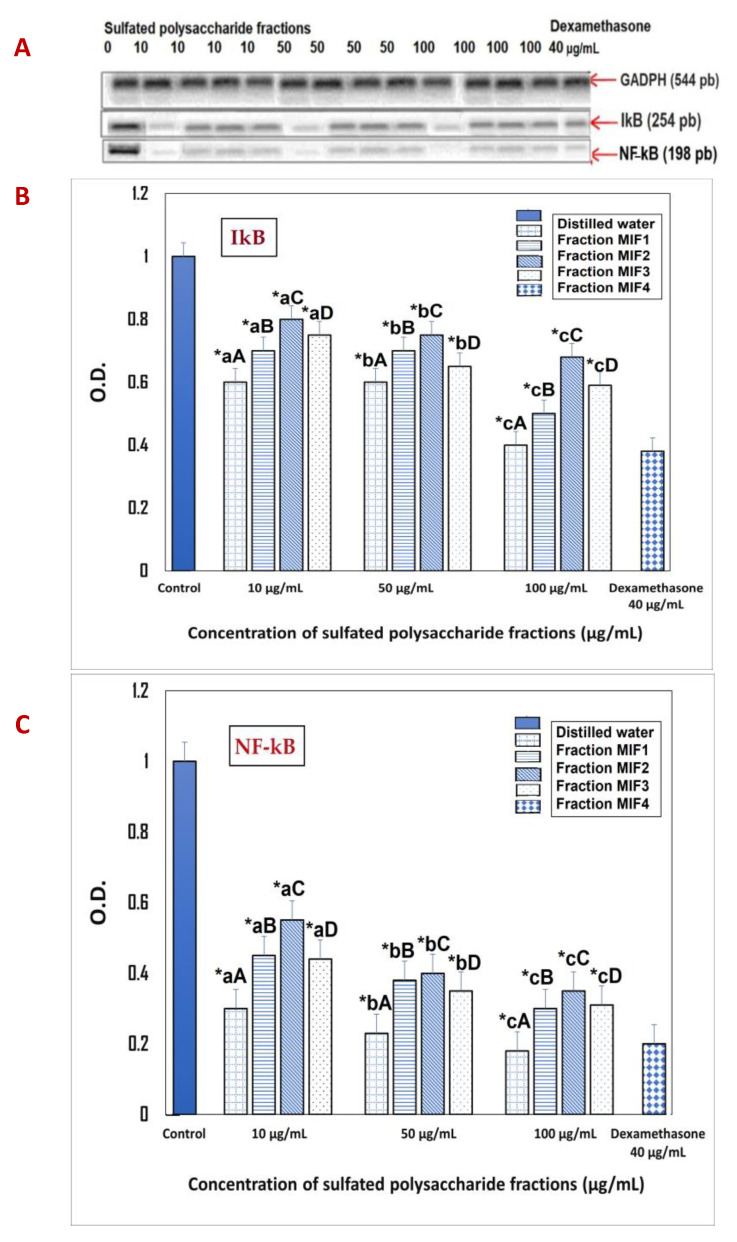
In vitro effects of *M*. *integrifolia* sulfated polysaccharides and dexamethasone on IκB and NF-κB mRNA levels in LPS- and IFNγ-stimulated murine peritoneal macrophages, as determined by RT-PCR. (**A**) analysis of the RT-PCR products on the 2% agarose gel; (**B**,**C**) IκB and NF-κB mRNA levels, respectively, quantified by densitometric analysis, expressed with respect to the parKa GADPH value obtained on the same gel. The bars show the mean ± SD (*n* = 3). * indicates statistically significant differences (*p* < 0.05) between the treatments +and the control, determined with the ANOVA statistic. Lowercase letters indicate statistical differences between the concentrations of the same fraction and uppercase letters indicate statistical differences between fractions (*p* < 0.05) with the Tukey multi-rank test.

Furthermore, the sulfated polysaccharides showed that, at the tested doses of 10, 50 and 100 µg/mL they inhibited the transcription of the genes encoding the NF-κB/IκB complex evaluated by RT-PCR, particularly MIF1. This revealed the highest inhibitory potential in all doses examined with 70%, 81% and 92% at 10, 50 and 100 µg/mL, respectively, with a *p* < 0.05. Likewise, M1F1 at 100 µg/mL showed a greater reduction compared to dexamatasone (40 µg/mL), which caused a reduction of 90% (Figure 8A–C). This indicated that sulfated polysaccharides inhibit the transcriptional activation of genes controlled by this transcription factor, most of which are involved in the immunoinflammatory response. It is important to note that the inhibition of NF-κB can be useful in the treatment of acute and chronic inflammatory diseases. Many anti-inflammatory therapies aim to block NF-κB activity; in this sense, an investigation using Fucoidan purified from *Fucus vesiculosus* exhibited anti-inflammatory activity by suppressing the NF-κB pathway, suggesting a down-regulation of pro-inflammatory mediators [57]. Furthermore, evidence has shown that when Toll-like receptors such as TLR2 and TLR4 are inhibited, there is a reduction in NF-κB transcriptional activities [58]; notably, the MyD88 gene, which provides instructions for making a protein involved in signaling within immune cells, showed a decrease in pro-inflammatory cytokine secretion and the NF-κB transcriptional activities of NF-κB [59].

### 2.7. Evaluation of M. integrifolia the Effect of Sulfated Polysaccharides on Allergic Response

#### 2.7.1. Ovalbumin (OVA) Induced Allergic Inflammation in Vivo Model

Ovalbumin is a glycoprotein in nature and constitutes the main protein components of the chicken egg whites. It is complex and large enough to elicit a mild immune reaction and, therefore, is commonly used as a T-cell-dependent antigen to trigger immunization in scientific research [60]. The results illustrated in Table 2 showed that the fractions tested for sulfated polysaccharides elicited a pronounced anti-inflammatory and anti-allergic behavior, as evidenced by the reveal of a significant reduction in plantar edema induced by Ovalbumin (OVA) at a dose of 100 mg/kg. The control group immunized with Ovalbumin (OVA) showed a marked increase in plantar edema, estimated at 95.6%, compared to the normal group that did not receive immunization. However, MIF1, MIF2, MIF3, and MIF4 treatment significantly reduced plantar edema, by 56, 27, 23 and 18%, respectively, compared to the ovalbumin immunized group. It is worth highlighting that MIF1 exerted anti-inflammatory and antiallergic behavior close to that of 5 mg/kg indomethacin, which caused a 57% reduction in ovalbumin-induced plantar edema. This is in accordance with an investigation that found that *Saccharina japonica* F-fucoidan showed an inhibitory effect on ear edema, finding that this effect depends on galectin-9, a suppressor of T and B cells, which prevents the binding of IgE to the IgE receptor FcεRI, inhibiting mast cell activation, which are regulatory cells of inflammatory and allergic processes [61].

#### 2.7.2. Histamine Skin Reaction

Histamine causes an increase in capillary and post-capillary venular permeability that ultimately leads to the wheal-flare response [62] reflected in the elevation of extravasated Evans blue in the histamine skin reaction assay. The results illustrated in Table 3 showed that tested fractions of sulfated polysaccharides also inhibited histamine release in vivo. Treatment with MIF1, MIF2, MIF3 and MIF4 significantly reduced histamine-induced skin reaction by 66.9%, 57.6%, 44.9% and 16.3%, respectively, with respect to the control group that received histamine solution only. However, promethazine treatment showed a 95.6% reduction in histamine-induced skin reaction (Table 3). In this context, the *Porphyra haitanensis* sulfated polysaccharide significantly inhibited histamine levels, also finding that IL-4, IL-5 and IL-13 levels decreased when co-cultured with the sulfated polysaccharide [63]. Furthermore, fucoidan from *Undaria pinnatifida* decreased histamine and IgE levels. In this sense, fucoidan suppresses Th-2 cytokines, such as IL-4, IL-5 and IL-13, as IgE production, and increases IgG2 production to suppress allergic reactions [64].

#### 2.7.3. Determination of Specific IgE in OVA-Immunized Mice

Additionally, the results illustrated in Table 4 showed that the fractions tested for sulfated polysaccharides caused a significant reduction in the serum IgE levels of OVA-immunized mice, as estimated by the passive cutaneous anaphylaxis test (PCA). Treatment with 50 mg/kg of MIF1, MIF2, MIF3, and MIF4 showed a notable reduction in serum IgE estimated by 54.4, 44.1, 41.0 and 43.0%, respectively, compared to the control group that receives only OVA, while ketotifen, at a dose of 3 mg/kg, showed a reduction of 66.2% in serum IgE with respect to the control group. Thus, it can be concluded that these compounds are capable of inhibiting the allergic inflammatory response, which coincides with a previous investigation [65]. In addition, low-molecular-weight fucoidan, which is rich in fucose and sulfate from *Laminaria japonica,* decreased allergen-specific IgE, proving that low-molecular-weight fucoidan had a significant effect on cytokine expression by enhancing the production of IL-2, IL-4 and IFN-γ, favoring TH1 differentiation, and decreases IgE, so these compounds may be useful in counteracting the allergic response because they act as adjuvants to enhance the immune-response-specific antigen [66].

## 3. Materials and Methods

### 3.1. Collection and Identification of Biological Material

Five kilograms of *Macrocystis integrifolia* algae were collected from the coastal waters of San Nicolás Bay, Marcona district, Ica region, Peru. To preserve algae during collection, the samples were immediately packed with ice and transported to the laboratory, where they were washed with distilled water, cut into small pieces and dried in a forced-air-circulation oven at 40 °C. Species identfication was carried out by the Herbarium Truxillense (HUT) with the deposit voucher HUT 25819.

### 3.2. Chemicals

In this investigation, all the chemicals used were of analytical grade and all were obtained from Sigma-Aldrich (St. Louis, MI, USA)).

### 3.3. Extraction of Sulfated Polysaccharides

One kilogram of dry ground seaweed was taken and extracted with distilled water at 55 °C, with continuous stirring for 4 h. The tissue was removed by simple filtration and the filtrate was centrifuged to remove the sediment until clear solutions were obtained. The clarified solution was precipitated with distilled ethanol and the solid was placed in an electric oven at 50 °C. Subsequently, the solid was subjected to fractionation by ion exchange chromatography, using a column (diameter = 2.2 cm, height = 20 cm) packed with 40 g of diethylaminoethylcellulose (DEAE-C), suspended in 1N Tris-HCl with a pH of 8.3. The extract rich in sulfated polysaccharides (1 g·15 mL^−1^) was dissolved in distilled water and incorporated into the column, using 5 volumes of NaCl (250 mL) at a concentration gradient of 0.5 M, 1.0 M and 2.0 M, and about 40 10 mL of eluates were collected. The eluates that showed a higher concentration was pooled and precipitated with three volumes of ethanol per 24 h. The supernatant was recovered by centrifugation (3000 rpm for 10 min), salt-purified by three consecutive washes with distilled water (10 mL), and precipitated again with three volumes of EtOH (30 mL). The supernatant of each fraction was recovered by centrifugation (3000 rpm for 10 min), dried in its entirety in an oven at 45 °C and stored in a vial [67,68,69]. Four fractions were obtained: MIF1 (350 mg), MIF2 (280 mg), MIF3 (400 mg), MIF4 (430 mg).

### 3.4. Fourier Transform Infrared (FTIR) Spectroscopic Analysis

Infrared spectra were obtained using a PerkinElmer TWO spectrophotometer equipped with a total reflectance (FTIR) attenuator. Each spectrum was taken in the spectral range of 400–4000 cm^−1^ [69]. Spectragryph optical spectroscopy software was used to process the spectra of the four evaluated fractions (MIF1, MIF2, MIF3 and MIF4).

### 3.5. Determination of the Chemical Composition of M. integrifolia Polysaccharide Fractions

The chemical composition of *M. integrifolia* polysaccharide fractions was investigated, where the composition of neutral sugar was determined by an HPLC assay after acid hydrolysis meanwhile the composition of uronic acid was determined by the carbazole method and calculated as the equivalent glucuronic acid [70]. Furthermore, the sulphate content of each fraction was determined by turbidimetric assay after acid hydrolysis [71], whereas the respective molecular weights of each polysaccharide fraction were determined using gel permeation chromatography [72].

### 3.6. Evaluation of Antiangiogenic Activity Using Chorioallantoic Membrane (CAM) Assay 

Sixty fertilized chicken eggs were used, obtained from the “Mochic” farm, which were washed, disinfected and incubated at 37 °C in a humidified atmosphere. On day 3, the upper surface of the egg was perforated, and 2 mL of albumin was extracted. This was then closed with cellophane paper, and a second 2.5 × 2 cm window was opened, exposing the underlying blood vessels. Bubbles generated by albumin suction were removed. Finally, the window was sealed with transparent tape and the recipients were returned to the incubator for one week. On day 10, a 5 mm diameter sterilized methylcellulose disc (MilliporeTM, Bedford, MA, USA) was placed on the surface of the CAM, in which the following solutions were placed: control group (*n* = 12): double-distilled water; the first group (*n* = 12): double distilled water and 100 µM of sulfated polysaccharide fraction (MIF1) 100 µM; the second group (*n* = 12): double-distilled water and 100 µM of sulfated polysaccharide fraction (MIF2); the third group (*n* = 12): double-distilled water and 100 µM of sulfated polysaccharide fraction (MIF3); the fourth group 4 (*n* = 12): double-distilled water and 100 µM of sulfated polysaccharide fraction (MIF4). On day 13, the MACs were removed and fixed in 10% formalin for 24 h, then subsequently washed with water and stained with hematoxylin–eosin (H-E). The microvascular density was determined by counting all blood vessels per 9000 µm^2^ (30 µm × 300 µm) in an optical microscope, with a magnification of 400× and an eyepiece located 600 µm from the filter. Counts were carried out in each of the work groups, obtaining sample values of the number of blood capillaries [73,74].

### 3.7. Determination of Cytotoxicity by MTT

Tetrazoyl-salt-reduction-based colorimetric technology (MTT) was used, where the secondary culture of chick embryo fibroblasts (CSFEP) was transferred to 96-well boxes (6000 cells per well) and incubated at 37 °C and 5% CO_2_ until a confluence of 80–90% was obtained. The medium was removed from the plates and 100 μL of polysaccharides per well were added at different concentrations: 1, 25, 50, 100, 200, 400, 600, 800 and 1000 μg/mL (three wells for each concentration). Three wells were left for control without sulfated polysaccharides; the assay was performed in triplicate. Plates were incubated for 48 h at 37 °C and 5% CO_2_. 0.025 g of the MTT reagent was prepared in 5 mL of PBS 1×, to obtain a concentration of 5 mg/mL of MTT solution. At the end of the incubation, the D-MEM/F12 medium was removed from the plates with CSFEP treated with sulfated polysaccharides, and 22 μL of the MTT solution was added to each well and incubated at 37 °C until a change in color was observed. A total of 100 μL of dimethylsulfoxide (DMSO) was added to solubilize the formazan salts in all wells, and incubation was performed for 8 min; then, the absorbance measurement was taken at 570 nm in the ELISA reader [73,74].

### 3.8. Evaluation of the Immunomodulatory Effect of Sulfated Polysaccharides from the Seaweed M. integrifolia on the Functionality of Peritoneal Macrophages

#### 3.8.1. Experimental Animals

Male Wistar rats (180–200 g) and Balb/c mice (male, 20–30 g) from the Animal Facility of the National Institute of Health, Lima, adapted for 7 days to laboratory conditions, were used. The animals were randomly distributed into groups of 5 animals and placed in a climate-controlled environment of 22 ± 1 °C and a 12 h cycle of light and darkness with a commercial diet for rodents and water ad libitum. The study was conducted under the international recommendations of the Declaration of the World Medical Association on the Use of Animals in Biomedical Research. The study was approved by the Ethics Committee of the Facultad de Farmacia y Bioquímica of the Universidad Nacional de Trujillo with the document COD. N°09: P-012-20/CEIFYB.

#### 3.8.2. Isolation of Peritoneal Macrophages for in Vitro Assays

Rats were sacrificed by cervical dislocation in a laminar flow chamber. A total of 30 mL of peritoneal fluid was removed. The cell suspension was washed by centrifugation at 400× *g* for 10 min at 4 °C and re-suspended in DSBH (Hank’s balanced salt solution). The cell concentration was adjusted to 106 cells/mL. The proportion of macrophages in the total population of peritoneal cells was determined by α-naphthyl staining [75,76].

#### 3.8.3. In Vivo Cellular Chemotaxis Assay

The experimental groups containing eight Wistar rats were arranged. To induce a chemotactic response towards the peritoneum of rats, 1 mL of 3% thioglycolate was injected intraperitoneally and 0.5 mL of sulfated polysaccharides was administered at doses of 10, 50 and 100 mg/kg in NaCl saline solution at 0.9%, corresponding to each experimental group. The control group received intraperitoneal saline solution. Five days later, the peritoneal exudate was collected, and the total number of cells was counted on a hemocytometer, determining the proportion of macrophages by staining with α-naphthyl [75,76].

#### 3.8.4. In Vitro Assay to Determine Phagocytic Activity

The phagocytic activity of macrophages was evaluated using the fluorimetric method based on the use of yeast (*Kluyveromyces lactis*), and marked with fluorescein isothiocyanate (ITCF) [75,76].

#### 3.8.5. In Vitro Assay for the Determination of Nitric Oxide Production

Nitric oxide production by peritoneal macrophages was determined by the Griess reaction by measuring the total amount of nitrites in the culture supernatant [75,76].

### 3.9. Determination of the Expression of Genes Encoding Enzymes, Cytokines and Immune Response Transcription Factors

#### 3.9.1. Collection and Activation of Murine Peritoneal Macrophages

Murine peritoneal macrophages were obtained from Balb/c mice and injected with 0.5 mL of 3% thioglycolate; then, after three days, the peritoneal exudate was obtained. The proportion of macrophages in peritoneal exudate was estimated by determining the nonspecific esterase activity [76,77].

#### 3.9.2. Determination of mRNA levels of NOS-2, COX-1, COX-2, NF-κB and IκB

Total RNA (tRNA) was isolated from macrophage samples and its quality was verified by conventional 2% agarose gel electrophoresis. The reverse transcription of the tRNA into complementary DNA (cDNA) was then carried out. cDNA synthesis (25 μL/reaction) was performed with 1.25 μM random hexamer primers, 250 mM of each deoxyribonucleotide triphosphate (dNTPs), 10 mM dithiotriethol (DTT), 20 U RNase inhibitor, 2, 5 mM MgCl_2_, 200 U murine leukemia virus reverse transcriptase in reaction-buffered solution (30 mM Tris and 20 mM KCl, pH 8.3), and 2 µg tRNA sample. The cyclic parameters for the reverse transcription reaction were hybridization for 10 min at 25 °C and reverse transcription for 60 min at 42 °C, by reverse transcription and polymerase chain reaction (RT-PCR). Polymerase chain reactions (PCR) were performed from 1 uL of the previous solution, using the following primers: NOS-2 (5′-TGGAAGCCGTAACAAAGGAAA-3′; 5′-ACCACTCGTACTTGGGATGCT-3′); COX-1 (5′-TGGTGGATGCCTTCTCTCG-3′; 5′-AACAGATGGGATTCCCTAGGA-3′); COX-2 (5′-TGATCGAAGACTACGTGCAAC-3′; 5′- TCATCTCTCTGCTCTGGTCAA-3′); NF-κB (5′-TAATCCTTGGGAGTGGAGCAA-3′; 5′-TGTTGGAGTGTGGCTGGAGT-3′); IκB (5′-ATTGCTGAGGCACTTCTGAA-3′; 5′-TTGACATCAGCACCCAAAGT-3′); GADPH (5′-ACCGCATCTTCTTGTGCAGT-3′; 5′- GCCAAAGTTGTCATGGATGA-3′). The amount of cDNA was quantified with the help of the Image Master densitometric analysis program (4.01, Amersham Pharmacia Biotech, Amersham, UK) [76,77].

### 3.10. Evaluation of the Effect of Sulfated Polysaccharides from M. integrifolia on the Allergic Response

#### 3.10.1. Ovalbumin (OVA) Induced Allergic Inflammation in Vivo Model

This was carried out using the in vivo model of allergic inflammation in seven experimental groups of eight Balb/c mice each, where six groups were immunized with Ovalbumin (OVA) in 0.5 mL of physiological saline. The remaining group only received injections of physiological saline solution. Three weeks after the last immunization, the groups were treated with saturated polysaccharides at doses of 50 mg/kg and 5 mg/kg of indomethacin, while the control group received distilled water. At the end of treatment, the allergic response was carried out using the ovalbumin-induced plantar edema model (OVA), and the proliferative response of splenocytes to OVA was determined [76,77].

#### 3.10.2. Histamine Skin Reaction

Six experimental groups of eight Wistar rats were arranged, who received treatment for 7 days with the administration of sulfated polysaccharides at doses of 50 mg/kg or promethazine at 25 mg/kg. Ten hours after the last administration, the rats were depilated in the dorsal region, drawing six quadrants of 1 × 1. In four of the quadrants, 20 mg/kg of Evans blue and 100 μL of saline solution with 5 μg of histamine were administered. In the remaining two, 100 μL of saline solution was administered. Thirty minutes later, the animals were sacrificed; the skin quadrants were cut and placed in test tubes with 2 mL of formamide and incubated at 37 °C for 48 h, determining the optical density of the transferred dye at 620 nm on a spectrophotometer [78,79].

#### 3.10.3. Determination of Specific IgE in OVA-Immunized Mice

Six experimental groups of eight Balb/c mice were organized and immunized with OVA according to the method described by Park et al. [80]. Then, for 21 days, mice were treated once daily with sulfated polysaccharides at concentrations of 50 mg/kg, or ketotifen (3 mg/kg). The control group only received distilled water. After 21 days, blood was extracted from mice, placed in test tubes, and centrifuged at 400× *g* for 10 min; serum was extracted and stored at −70 °C for later use. Subsequently, to estimate the levels of a specific IgE, the passive cutaneous anaphylaxis test (PCA) was performed in Wistar rats using the antiserum obtained in mice immunized with OVA and treated with sulfated polysaccharides or ketotifen.

### 3.11. Statistical Analysis

Data were presented as mean ± standard deviation (SD). The ANOVA test was performed to determine the differences between groups. In addition, a Tukey multirank test was used to indicate differences between the concentrations of the same fraction, as well as the differences between fractions. All statistical analyses were performed using SPSS v.25.0 (IBM Corp., Armonk, NY, USA), and Graphpad Prism 6.0^®^ (GraphPad Software, San Diego, CA, USA) 4. Conclusions

From the current study, it was concluded that *M. integrifolia* algae are a rich source of sulfated polysaccharides, as revealed by Fourier transform infrared (FTIR) spectroscopic analysis. All tested fractions of sulfated polysaccharides, particularly MIF1, showed notable antiangiogenic activity using the chorioallantoic membranes (CAM) assay. They also showed potent immunomodulatory potential, as evidenced by the inhibition of chemotaxis, phagocytosis, and mRNA expression of NOS-2, COX-1, COX-2, NF-κB and IκB. Additionally, they showed antiallergic potential in ovalbumin (OVA)-induced allergic inflammation in an in vivo model, with concomitant reductions in histamine-induced skin reaction and specific IgE in OVA-immunized mice. The biological activities of sulfated polysaccharides are intimately dependent on the chemical structure and molecular weight, as well as the extraction process; thus, low-molecular-weight sulfated polysaccharides showed better pharmacological activities than high-molecular-weight ones. In addition, the content of the fucose and sulfate groups could influence immunomodulatory activity. In the same way, antiangiogenic activity is related to sulfated polysaccharides with MW > 30 kDa; however, a low molecular weight promotes angiogenesis. Therefore, sulfated polysaccharides and their chemical characteristics may explain the observed activity. This means that *M. integrifolia* sulfated polysaccharides could serve as promising, naturally occurring, antiangiogenic and immunomodulatory drug entities that could be incorporated into pharmaceutical dosage forms to combat various immune-related disorders. However, further preclinical studies should be conducted to consolidate the obtained results. 

## Figures and Tables

**Table 1:** Chemical composition of the fractions obtained from *Macrocystis integrifolia.*

Fraction	Composition of Neutral Sugar (%) ^a^						UA (%) ^b^	SO_4_^2−^ (%) ^c^	Mw (kDa) ^d^
	Fuc	Xyl	Man	Gal	Glu	Rha			
M1F1	88.5	3.6	4.1	1.2	1.5	1.1	3.8	33.2 ^a^	49.2 ^a^
MIF2	88.1	3.8	4.9	1.8	0.6	0.8	5.1	27.2 ^b^	62.1 ^b^
MIF3	87.1	-	-	12.9	-	-	4.8	26.8 ^c^	70.8 ^c^
MIF4	100	-	-	-	-	-	4.1	25.1 ^d^	88.4 ^d^

^a^ Determined by an HPLC assay after acid hydrolysis, ^b^ determined by the carbazole method and calculated as the equivalent glucuronic acid, ^c^ determined by turbidimetric assay after acid hydrolysis, and ^d^ determined by gel permeation chromatography; different letters indicate statistical differences (*p* < 0.05) (*n* = 3).

**Table 2:** Effect of pretreatment for seven days with the fractions of sulfated polysaccharides and indomethacin on plantar edema induced by OVA in immunized mice.

Treatments	Dose (mg/kg)	Oedema Index (g)	% Inhibition of Inflammation and Allergy
Normal (No immunization)	-	18 ± 0.41	-
Control	-	53.2 ± 1.50	-
MIF1	50	23.2 ± 1.10	56
MIF2	50	38.9 ± 1.50	27
MIF3	50	41.2 ± 1.50	23
MIF4	50	43.8 ± 1.50	18
Indomethacin	5	22.9 ± 0.80	57

**Table 3:** Effect of pretreatment for seven days with fractions of sulfated polysaccharides and promethazine on histamine-induced skin reaction in rats.

Treatments	Dose (mg/kg)	Extravasated Evans Blue (µg/site)	% Inhibition of Skin Reaction
Control		24.5 ± 0,41	-
MIF1	50	8.1 ± 1.2	66.9
MIF2	50	10.4 ± 1.1	57.6
MIF3	50	13.5 ± 0.8	44.9
MIF4	50	20.5 ± 1.2	16.3
Promethazine	25	1.1 ± 0.16	95.6

**Table 4:** Effect of treatment for 21 days with sulfated polysaccharide fractions and ketotifen on serum IgE levels of OVA-immunized mice, determined by passive cutaneous anaphylaxis.

Treatments	Dose (mg/kg)	Extravasated Evans Blue (g/site) Serum Dilution 1/256	% Inhibition of Skin Reaction
Control		6.8 ± 0.41	-
MIF1	50	3.1 ± 0.9	54.4
MIF2	50	3.8 ± 0.8	44.1
MIF3	50	4.0 ± 0.9	41.0
MIF4	50	3.9 ± 1.2	43.0
Ketotifen	3	2.3 ± 0.20	66.2

## Data Availability

Data are available in the manuscript.
